# Successful conversion therapy for presumed granulocyte colony-stimulating factor–producing hepatocellular carcinoma: a case report

**DOI:** 10.3389/fonc.2026.1808263

**Published:** 2026-06-03

**Authors:** Qian Chen, Taian Chen, Xu Zhang, Ming Zhang

**Affiliations:** 1 Department of Hepatobiliary and Pancreatic Surgery, Yibin First People’s Hospital, Yibin, China; 2 Department of Pathology, Yibin First People’s Hospital, Yibin, China

**Keywords:** granulocyte colony-stimulating factor, hepatocellular carcinoma, immunotherapy, targeted therapy, transcatheter arterial chemoembolization

## Abstract

**Introduction:**

Granulocyte colony-stimulating factor (G-CSF) is an endogenous glycoprotein that is classically known to be important in the proliferation of hematopoietic progenitor cells and the differentiation of neutrophils. In recent years aberrant G-CSF expression has been described in various malignancies and is believed to play a role in tumor progression, however, G-CSF-producing hepatocellular carcinoma (HCC) is extremely rare. Here, we describe a rare case of locally advanced presumed G-CSF-producing HCC. To the best of our knowledge, this is one of the few reported cases of a patient with presumed G-CSF-producing HCC who did not exhibit any specific clinical symptoms and was successfully operated upon after undergoing conversion therapy.

**Case report:**

The patient had no symptoms but a massive tumor in the right hepatic lobe accompanied by significant leukocytosis. The lesion was originally considered unresectable. Following combination therapy with transcatheter arterial chemoembolization (TACE) in conjunction with targeted therapy and immunotherapy, the tumor was successfully downstaged to a resectable state. The patient tolerated the combined regimen well without significant adverse events, and curative-intent surgical resection was performed. Postoperative immunohistochemical analysis revealed strongly positive expression of G-CSF in the HCC tissue, supporting the presumptive diagnosis of G-CSF-producing HCC, although pre-treatment serum G-CSF levels were not measured. G-CSF-producing HCC is rare and is usually linked to aggressive behavior and poor prognosis. In this case, however, no recurrence was seen at 6-month follow-up and the patient was in good general condition.

**Conclusions:**

Clinicians should suspect G-CSF-producing HCC in patients with HCC who have unexplained leukocytosis. For those individuals who are not candidates for upfront resection, a combined approach using TACE, targeted therapy and immunotherapy may be a feasible therapeutic option, although further studies are needed to validate this approach.

## Introduction

Granulocyte colony-stimulating factor (G-CSF) is an endogenous glycoprotein that is traditionally thought to be a major regulator of the hematopoietic system. In the past few years aberrant G-CSF expression has been identified in various types of malignancies and has been implicated in tumor biology, especially in lung and breast cancers (1, 2). However, reports of G-CSF-producing hepatocellular carcinoma (HCC) are extremely rare. Accumulating evidence indicates that dysregulated G-CSF expression is linked to aggressive tumor behavior and poor prognosis; however, the exact mechanisms are not yet fully understood and the optimal therapeutic strategies have not yet been determined (3). In this study, we report a rare case of locally advanced G-CSF-producing HCC that was successfully downstaged by the use of a combined regimen of transcatheter arterial chemoembolization (TACE), targeted therapy and immunotherapy, ultimately allowing for curative-intent surgical resection. This case offers practical insight into the possible management of similar patients in clinical practice.

## Case report

A 61-year-old man was admitted to our hospital on March 5, 2025, due to a recently discovered space-occupying lesion in the liver. He denied any significant past medical history, and reported no chills, fever or other specific discomfort. Physical examination on admission was unremarkable. The abdomen was soft, non-tender, and non-distended, with no palpable hepatomegaly. There were no stigmata of chronic liver disease, including no spider angiomata, palmar erythema, jaundice, or caput medusae. No ascites was detected. Laboratory evaluation showed white blood cell count (WBC) of 23.89 × 109/L and absolute neutrophil count (NEUT) of 21.98 × 109/L. Alpha-fetoprotein (AFP) was negative while hepatitis B surface antigen (HBsAg) was positive. Dynamic magnetic resonance imaging (MRI) revealed a mass in the right hepatic lobe with a maximal cross-sectional diameter of 13.5 × 7.9 cm. Enhancement of the mass showed a classical “wash-in and wash-out” pattern, favoring the diagnosis of HCC, and the portal vein was well visualized (**Figure 1**). Contrast-enhanced computed tomography (CE-CT) showed a lobulated hypodense lesion in the right hepatic lobe measuring approximately 14.0 × 11.1 cm with moderately heterogeneous arterial phase enhancement and non-enhancing hypodense necrotic component within the tumor, consistent with HCC (**Figure 2**). Based on these results, the patient was diagnosed with HCC (CNLC stage IIb), with hepatic abscess in the differential diagnosis. Liver function was Child-Pugh class A (6 points) and Eastern Cooperative Oncology Group (ECOG) performance status was 0. Given the initial suspicion of concomitant hepatic abscess, empiric anti-infective therapy with cefoperazone-sulbactam and ornidazole was initiated for one week. Procalcitonin (PCT) was 0.6 ng/mL and blood cultures were negative, which did not support a significant bacterial infection. However, after one week of treatment, repeat laboratory testing showed further increases in WBC to 28.63 × 109/L and NEUT to 26.96 × 109/L. In the face of the absence of response to antibiotics, the leukocytosis and neutrophilia were thought to be noninfectious and most consistent with a tumor-associated leukemoid reaction; thus, hepatic abscess was ruled out. Notably, the subsequent normalization of WBC and NEUT counts in parallel with tumor treatment response further supported a tumor-related etiology and argued against a primary hematologic disorder. Due to the high tumor burden and insufficient future liver remnant (FLR of less than 30%, as assessed by three-dimensional reconstruction using Myrian software), a combination regimen was chosen, TACE plus targeted therapy and immunotherapy (bevacizumab 800 mg and sintilimab 200 mg every 21 days), combined with antiviral therapy (entecavir 0.5 mg once daily). Given the large tumor size and to minimize the risk of post-procedural hepatic abscess, staged TACE was adopted at our center.

**Figure 1:**
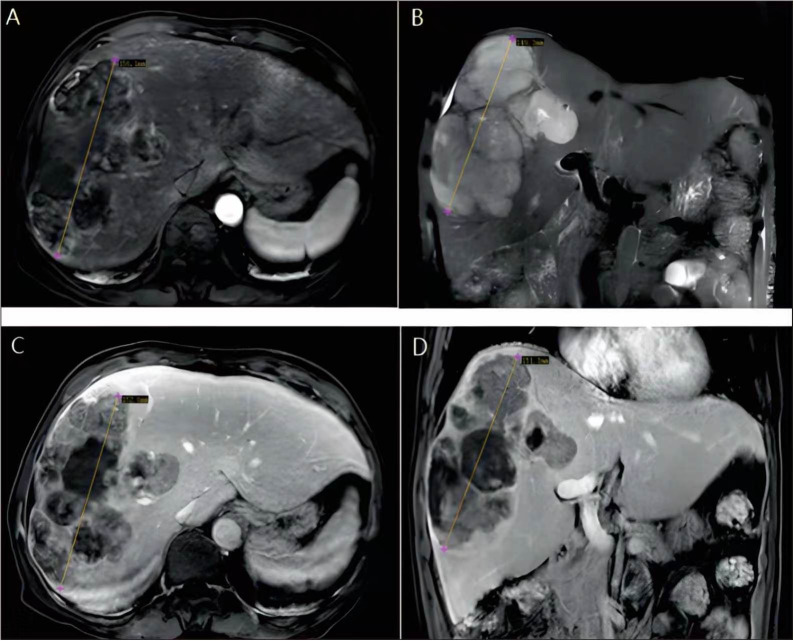
Pre-treatment dynamic magnetic resonance imaging showing a 13.5 × 7.9 cm mass in the right hepatic lobe. **(A)** arterial phase; **(B)** unenhanced phase; **(C, D)** portal venous phase.

**Figure 2:**
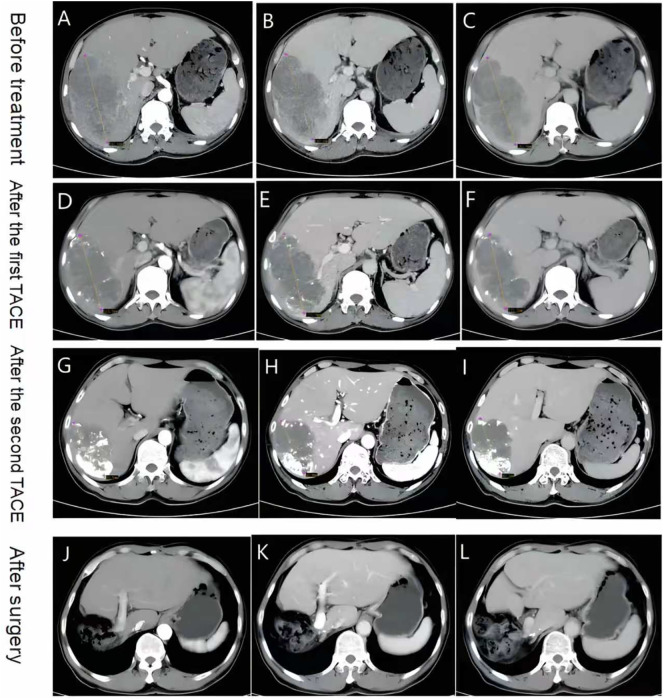
CE-CT dynamic changes before and after treatment. **(A–C)** Pre TACE: 13.5 × 7.9 cm mass in the right hepatic lobe, highly suggestive of HCC. **(D–F)** After the first TACE: minimal lipiodol retention in the lesion with increased intralesional necrosis. **(G–I)** After the second TACE: extensive lipiodol deposition with further tumor necrosis and tumor shrinkage. **(J–L)** Six months after surgical resection: no recurrence.

The patient had the first session of TACE on March 13, 2025. Laboratory testing on postoperative days 1, 3, and 5 showed a gradual decrease in both the WBC count and absolute NEUT (**Figure 3**). The postoperative course was uneventful without chills, fever, or other discomfort and the patient was discharged as scheduled. Approximately 40 days later, a second TACE procedure was performed on April 24, 2025. Preprocedural laboratory evaluation revealed additional decreases in WBC and NEUT when compared with previous measurements (**Figure 3**). Contrast-enhanced CT showed widespread intratumoral necrosis (**Figure 2**). Postprocedural laboratory results showed that both WBC and NEUT had returned mostly to the normal range (**Figure 3**).

**Figure 3:**
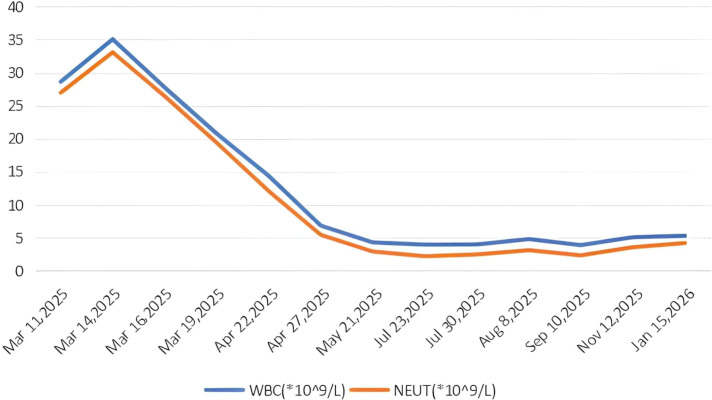
Dynamic changes in WBC and NEUT counts from baseline to January 2026.

After two TACE sessions and four rounds of systemic therapy, CE-CT done on July 23, 2025, showed significant tumor necrosis with a decrease in lesion size compared to previous imaging (**Figure 2**). According to the modified Response Evaluation Criteria in Solid Tumors (mRECIST), as independently assessed by a radiologist, the maximum diameter of viable (arterial-enhancing) tumor decreased from 15.5 cm at baseline to 3.2 cm, meeting the criteria for partial response. Reassessment by CT volumetry using Myrian software revealed reduced tumor burden and a sufficient future liver remnant of more than 40%, and successful conversion of the disease from unresectable to resectable. Throughout the course of TACE and systemic therapy, the patient tolerated the combined regimen well, with no significant treatment-related adverse events observed. No dose modifications or treatment delays were required, and the patient completed all planned treatment cycles. After a 4-week washout period from targeted therapy and immunotherapy, curative-intent surgery was performed on August 1, 2025. The patient recovered uneventfully and was discharged as planned. Gross examination of the resected specimen showed a tumor measuring about 12.0 × 8.5 cm. Histopathological analysis showed extensive tumor necrosis with residual viable tumor of <5%, with no evidence of microvascular invasion, satellite nodules, or cholangiocarcinoma components. Surgical margins were negative, with a clearance of more than 1 cm, confirming R0 resection. Immunohistochemistry showed the following profile: Vimentin (+), CD10 (+), GS (partially +), Ki-67 (positive, approximately 40% in hotspot areas), PCK (−), EMA (−), CK8/18 (−), HepPar-1 (−), Glypican-3 (−), Arginase-1 (−), AFP (−), CK19 (−), CD34 (−), Desmin (−), S100 (−), HMB45 (−), Melan-A (−), CK5/6 (−), P63 (−), and P40 (−). The loss of classic hepatocyte markers (HepPar-1, Glypican-3, and Arginase-1) combined with Vimentin positivity was consistent with poorly differentiated HCC with sarcomatoid differentiation. It should be noted that this immunohistochemical pattern could also raise the possibility of other diagnoses, including primary hepatic sarcoma, undifferentiated carcinoma, or metastatic tumors. However, several factors supported the diagnosis of sarcomatoid HCC in this case: (1) the tumor arose within the liver in a patient with chronic hepatitis B, a well-established risk factor for HCC; (2) CD10 positivity, which has been reported in a subset of HCC cases; (3) GS partial positivity, which is a marker associated with hepatocellular differentiation; (4) the absence of markers suggestive of other lineages, including S100, HMB45, Melan-A (melanoma), Desmin (mesenchymal tumors), CK5/6, P63, P40 (squamous differentiation), and CK19 (cholangiocarcinoma); and (5) the clinical context, imaging characteristics, and hepatitis B background were all consistent with a primary hepatic malignancy rather than a metastatic lesion. G-CSF staining was strongly positive, with nearly 100% of viable tumor cells demonstrating uniform cytoplasmic brown granular staining, which was clearly distinguishable from the negative control. G-CSF staining was evaluated exclusively in viable tumor areas to avoid non-specific staining in necrotic regions (**Figure 4**). Post-operatively, the patient was kept on maintenance therapy based on the original systemic regimen and followed regularly. At six months after surgery, follow-up examination showed no increase in white blood cell (WBC) and neutrophil (NEUT) counts (**Figure 3**), and contrast-enhanced computed tomography (CE-CT) showed no recurrence of the tumor (**Figure 2**). The detailed clinical timeline is presented in **Table 1**.

**Figure 4:**
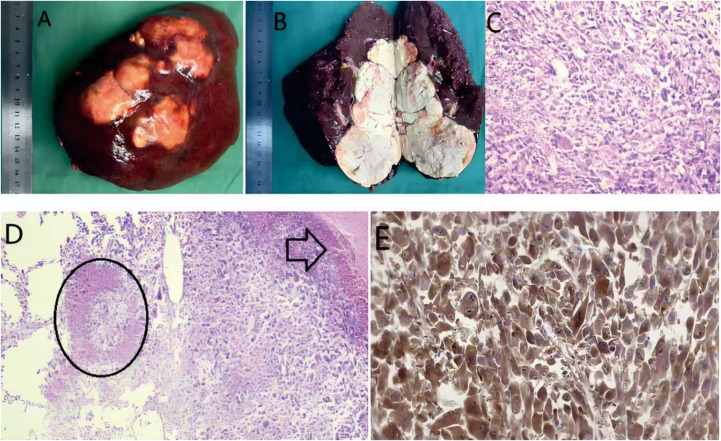
Pathological gross specimen and immunohistochemical examination of the tumor. **(A)** Liver specimen with a protruding tumor. **(B)** Section of the specimen showing coagulative tumor necrosis. **(C)** Viable tumor cells with significant pleomorphism (poorly differentiated HCC) (hematoxylin-eosin staining, 10×10). **(D)** Viable and necrotic tumor cells, showing marked tumor necrosis (hematoxylin-eosin staining, 10×10). **(E)** Immunohistochemical staining (EnVision two-step method) revealing diffuse strong expression of G-CSF in viable tumor cells, with a positive cell ratio of nearly 100% (10×20).

**Table 1:** Clinical timeline.

Date	Clinical event	Key findings
March 5, 2025	Hospital admission	Liver mass detected; WBC 23.89 × 10^9^/L, NEUT 21.98 × 10^9^/L; AFP (−), HBsAg (+); PCT 0.6 ng/mL; blood cultures negative
March 5, 2025	Imaging assessment	MRI: right hepatic lobe mass, 13.5 × 7.9 cm, “wash-in and wash-out” pattern; CE-CT: 14.0 × 11.1 cm lobulated lesion with heterogeneous enhancement
March 5, 2025	Diagnosis	HCC (CNLC stage IIb); Child-Pugh A (6 points); ECOG PS 0; FLR < 30% (Myrian software), deemed unresectable
March 5–12, 2025	Empiric anti-infective therapy	Cefoperazone-sulbactam + ornidazole for 1 week; no clinical response; WBC increased to 28.63 × 10^9^/L, NEUT to 26.96 × 10^9^/L; hepatic abscess ruled out
March 13, 2025	First TACE session	Staged TACE performed; postoperative WBC and NEUT gradually decreased
March 2025	Initiation of systemic therapy	Bevacizumab 800 mg + sintilimab 200 mg every 21 days; entecavir 0.5 mg daily
April 24, 2025	Second TACE session	Further decrease in WBC and NEUT; CE-CT showed widespread intratumoral necrosis; WBC and NEUT returned mostly to normal range
July 23, 2025	Conversion assessment	CE-CT showed significant tumor necrosis and decreased lesion size; mRECIST: viable tumor diameter decreased from 15.5 cm to 3.2 cm (partial response); FLR > 40%; successful downstaging confirmed
August 1, 2025	Curative-intent surgical resection	R0 resection; margin > 1 cm; gross specimen: 12.0 × 8.5 cm; pathology: extensive necrosis, viable tumor < 5%, no MVI, no satellite nodules; IHC: G-CSF strongly positive, poorly differentiated HCC with sarcomatoid differentiation
February 2026	6-month follow-up	No recurrence on CE-CT; WBC and NEUT within normal range; patient in good general condition

## Discussion

In this report, we present a case of G-CSF-producing HCC. The patient was not a candidate for surgical resection initially, but after TACE combined with systemic therapy, the tumor burden was reduced substantially and successful curative-intent resection was performed. To the best of our knowledge, this is one of the few reported cases of a patient with presumed G-CSF-producing HCC who did not exhibit any specific clinical symptoms and was successfully operated upon after undergoing conversion therapy.

In the last years, accumulating evidence from a wide variety of solid tumors has led to the hypothesis that overexpression of G-CSF is linked to increased tumor aggressiveness and poor prognosis (4). Nevertheless, G-CSF-producing HCC is extremely rare, with few cases reported so far. G-CSF-producing tumors are usually characterized by the following features (5): (1) marked leukocytosis; (2) a decrease in leukocyte counts after resection of the tumor; and (3) positive G-CSF staining on immunohistochemistry of tumor tissue. While the diagnosis was not suspected at the time of initial disease, we did not measure pre-treatment serum granulocyte colony-stimulating factor (G-CSF) levels. However, the patient showed elevated leukocyte counts at baseline, which normalized after treatment. When combined with the immunohistochemical results, these data are suggestive of G-CSF-producing hepatocellular carcinoma (HCC). We acknowledge that the absence of pre-treatment serum G-CSF measurement is a limitation of this case, and the diagnosis of G-CSF-producing HCC remains presumptive rather than definitive. However, the triad of marked leukocytosis at baseline, normalization of leukocyte counts following tumor treatment, and strongly positive G-CSF immunohistochemical staining in tumor tissue is consistent with the diagnostic criteria proposed in the literature. In previously published reports (5–9), patients with G-CSF-producing HCC usually had systemic manifestations such as fever and malaise, resembling an infectious or toxemic state. By contrast, the current patient had no prominent symptoms, representing a different clinical phenotype compared with previous cases. This unusual presentation caused diagnostic problems and highlights the fact that fever and associated systemic symptoms are not mandatory features of G-CSF-producing HCC, and differential diagnosis should therefore be done carefully.

Previous studies have shown that G-CSF is abnormally expressed at high levels in several solid tumors, such as lung and colorectal cancers, and that this high expression is linked to rapid tumor growth and poor clinical outcomes (10, 11). Consistently, patients with G-CSF-producing HCC tend to have an aggressive biological behavior and an unfavorable prognosis. A summary of previously reported cases of G-CSF-producing HCC is presented in **Table 2**. In previous reports (5–9), the longest overall survival of patients with G-CSF-producing HCC in the early stages who underwent curative resection was up to 4 years. By contrast, most reported cases of intermediate- to advanced-stage G-CSF-producing HCC resulted in death within 6 months, despite active treatment, including attempts at curative-intent surgery. Moreover, available evidence suggests that sarcomatoid transformation is associated with an even worse prognosis in G-CSF-producing HCC (5). Accordingly, early detection and early surgical intervention are important for better outcome and survival in this rare entity. However, optimal management of intermediate- to advanced-stage G-CSF-producing HCC, especially in patients with concomitant sarcomatoid differentiation, is very difficult.

**Table 2:** Summary of previously reported cases of G-CSF-producing HCC.

Study	Stage	Treatment	Outcome
Araki et al. (6)	Early	Curative resection	Longest survival up to 4 years
Joshita et al. (7)	Early	Curative resection	Survived
Kohno et al. (8)	Intermediate to advanced	Curative-intent surgery	Died within 6 months
Nagata et al. (9)	Intermediate to advanced	Active treatment	Died within 6 months
Sakamoto et al. (5)	Intermediate to advanced	Resection + chemotherapy	Died within 6 months
**Present case**	**Intermediate (CNLC IIb)**	**TACE + bevacizumab + sintilimab → curative resection**	**No recurrence at 6 months; OS approaching 1 year**

Bold values indicate the present case reported in this manuscript, to distinguish it from previously published cases.

The majority of patients with HCC are diagnosed at intermediate- and advanced-stage disease, thus missing the chance for surgical treatment and showing poor survival rates. Over the last few years, the rapid development of interventional therapy, targeted therapy and immunotherapy has encouraged researchers to consider conversion therapy, which involves local and systemic therapies to convert unresectable HCC to resectable HCC, and many such cases have been reported (12–14). In this case, the patient was initially diagnosed with CNLC stage IIb HCC. The large size of the tumor and the estimated future liver remnant of less than 30% prevented immediate surgery. In line with the guidelines (15), the combination of bevacizumab and sintilimab represents a first-line systemic therapy for unresectable HCC, and the addition of interventional therapy can enhance the therapeutic effect and survival rate. Considering the poor prognosis of intermediate or advanced G-CSF-producing HCC reported in the previous studies (5–9), we adopted a multimodal approach involving TACE in combination with systemic therapy, to achieve good prognosis and a surgical opportunity via successful downstaging. The patient showed partial remission by the modified Response Evaluation Criteria in Solid Tumors (mRECIST) (16) after two cycles of TACE and four cycles of systemic therapy. The reduction in tumor size and the estimated future liver remnant of more than 40% allowed for successful downstaging. Following recommendations from relevant studies (17), R0 surgical resection was performed four weeks after drug withdrawal. There was no tumor recurrence at the six-month follow-up, which is an encouraging outcome compared with previously reported intermediate or advanced G-CSF–producing HCC cases, although longer follow-up is needed to confirm its durability (5, 6, 8), and can be attributed to the successful conversion therapy. In addition, a significant degree of tumor necrosis observed in the postoperative pathological report also verified the effectiveness of this strategy.

Until recently, there were few treatment options for intermediate or advanced G-CSF-producing HCC, which explains the poor survival rate seen in these patients (5, 6, 8). The advent of targeted and immunotherapies has revolutionized treatment of intermediate and advanced HCC. Sintilimab, an immune checkpoint inhibitor, activates inhibited T cells, thereby increasing their ability to recognize and kill tumors, and is commonly used for the treatment of intermediate and advanced HCC in China (18). Furthermore, immunohistochemical staining of tumor tissues from four patients with G-CSF–producing HCC revealed expression of programmed death-ligand 1 (PD-L1) in all four patients, suggesting a response to immunotherapy (19). Bevacizumab is a recombinant humanized monoclonal antibody that blocks vascular endothelial growth factor (VEGF), reducing angiogenesis and tumor growth (20). There is evidence that bevacizumab and sintilimab synergistically modulate the tumor immune microenvironment, with a greater impact than the simple additive effects of each (21). As such, this regimen is considered a first-line systemic treatment and is commonly used in China for intermediate and advanced HCC. TACE also augments this therapy. Although TACE-induced ischemia increases VEGF, which stimulates angiogenesis and may decrease TACE’s effectiveness, TACE-induced tumor necrosis also promotes the release of tumor antigens and activates the immune response against the tumor. Thus, the combination of TACE with sintilimab and bevacizumab may have a greater-than-additive effect, leading to improved efficacy and survival outcomes for intermediate and advanced HCC (22). In this case, the combination of TACE, sintilimab and bevacizumab successfully downstaged the tumor and allowed for surgical resection. However, as this is a single case treated with a multimodal approach, it is not possible to isolate the contribution of each individual component to the observed outcome. Nevertheless, this case may serve as a reference for the management of patients with intermediate and advanced G-CSF–producing HCC. It should also be noted that the treatment regimen employed in this case represents a standard-of-care approach for advanced HCC, rather than a strategy specifically targeting G-CSF-mediated tumor biology. To date, no targeted therapies directed against G-CSF signaling have been established for G-CSF-producing HCC. Future studies exploring the role of G-CSF receptor inhibition or other G-CSF-directed interventions in this rare subtype are warranted.

This case has several limitations that should be acknowledged. First, as mentioned above, pre-treatment serum G-CSF levels were not measured because the diagnosis of G-CSF-producing HCC was not initially suspected. Furthermore, serum G-CSF measurement was not available at our institution due to limited laboratory testing capabilities, which precluded both pre- and post-operative serum G-CSF quantification. Therefore, the diagnosis remains presumptive. Second, the exclusion of other causes of leukocytosis was not exhaustive. Ideally, a comprehensive workup should have included C-reactive protein (CRP), peripheral blood smear, and bone marrow examination to more rigorously exclude infectious etiologies and primary hematologic disorders such as chronic myeloproliferative neoplasms. In this case, CRP was not measured and bone marrow examination was not performed, which represents a notable limitation in the diagnostic workup. Nevertheless, the normalization of WBC and NEUT counts in parallel with tumor treatment response and their sustained normal levels postoperatively strongly suggest a tumor-related etiology rather than a primary hematologic disorder or chronic infection. Third, the postoperative follow-up period of six months is a major limitation of this study, particularly given the well-documented aggressive biology of G-CSF-producing HCC and its high propensity for early recurrence. The favorable outcome observed in this case should therefore be interpreted with considerable caution, as it does not preclude the possibility of late recurrence. Nevertheless, the patient’s overall survival has reached one year at the time of writing, which exceeds the outcomes reported in previous cases of intermediate to advanced G-CSF-producing HCC, in which most patients died within six months despite active treatment. We will continue to follow this patient closely and report long-term outcomes in due course. Finally, as this is a single case report treated with a multimodal approach combining TACE, bevacizumab, and sintilimab, the individual contributions of each therapeutic component to the tumor response and successful downstaging cannot be determined. The findings therefore cannot be generalized, and no definitive therapeutic conclusions should be drawn from this case alone. Prospective studies or larger case series are needed to validate this approach and to identify the optimal treatment strategy for this rare tumor subtype.

In summary, G-CSF-producing HCC should be considered in patients with HCC who have an unexplained leukocytosis, even in the absence of fever. Nevertheless, the pathophysiological mechanisms that contribute to this are still to be fully elucidated. For patients with early-stage G-CSF-producing HCC, early surgical resection may significantly improve the prognosis. For intermediate to advanced stage disease, an integrated approach that includes TACE, targeted therapy and immunotherapy may be a feasible therapeutic option and could potentially provide opportunities for successful conversion into resectability, although further studies are needed to confirm these findings. Further studies and additional clinical cases are required to further elucidate the pathogenesis, clinical features and optimal management strategies for G-CSF-producing HCC.

### Patient’ s perspective

The publication of this article and the chart obtained the informed consent of the patients. The patients are very satisfied with the curative effect of the treatment, full of confidence in their future life and fully affirmed our work.

## Data Availability

The original contributions presented in the study are included in the article/supplementary material. Further inquiries can be directed to the corresponding author.
